# A comment on ‘Efficacy of mitral valve repair in combination with coronary revascularization for moderate ischaemic mitral regurgitation: a systematic review and meta-analysis of randomized controlled trials’

**DOI:** 10.1097/JS9.0000000000001508

**Published:** 2024-05-03

**Authors:** Jiayun Zheng, Zeru Chen

**Affiliations:** a The First Clinical Medical School, Guangzhou Medical University; b The Second Clinical Medical School, Guangzhou Medical University, Guangzhou, Guangdong, People’s Republic of China

Ischemic mitral regurgitation (IMR) is a growing concern in the field of cardiac disease, with its occurrence being closely related to cardiac events and patient prognosis. According to reports, more than 10% of individuals experience IMR after myocardial infarction^1^. This highlights the importance of adequate management and treatment approaches for this illness. Beyond its noteworthy effect on improving heart function, mitral valve repair (MVR) has attracted interest because of its possible advantages in symptom relief and patient quality of life. New data suggests that MVR plus coronary artery bypass grafting (CABG) may be beneficial in treating moderate IMR, providing patients with a wider range of treatment alternatives.

The article ‘Efficacy of mitral valve repair in combination with coronary revascularization for moderate ischaemic mitral regurgitation: a systematic review and meta-analysis of randomized controlled trials^2^’ conducted by Li *et al*. caught our attention recently. In randomized controlled trials, they examined the effectiveness of MVR in conjunction with CABG for the management of moderate IMR. Their results indicate that, as compared to CABG alone, MVR combined with CABG did not significantly raise the risk of postoperative stroke, worsening renal function (WRF), and reoperation for bleeding or pericardial tamponade. The strongest evidence on the appropriateness of performing MVR during CABG in patients with moderate IMR can be found in these findings. Even though this research offers valuable information for clinical treatment, several areas still require discussion and improvement.

Firstly, the primary outcomes of the study focused on operative mortality and long-term mortality, with secondary outcomes including postoperative stroke, WRF, and reoperation for bleeding or embolism. The study concluded that MVR combined with CABG did not significantly increase these risks compared to CABG alone. I would advise the study to take into account additional potential complications of the combination surgery, such as infection, myocardial damage, atrial fibrillation, and valve malfunction. In addition, it is recommended to include long-term follow-up studies to more thoroughly assess the long-term effects of surgery. Indicators such as NYHA classification, SF-36, or MLHFQ questionnaires, and cardiac ultrasound index can be used to assess patients’ symptoms, quality of life, and cardiac function.

Secondly, the subgroup analysis of the study observed that in patients with LVEF (left ventricular ejection fraction) <40%, CABG combined with MVR demonstrated a superior prognosis in terms of long-term mortality compared to CABG alone. But, I would argue that the analysis should also take into account the variations in how gender affects surgery. Research indicates that following open cardiac valve surgery, female patients have an estimated 41% higher risk of in-hospital mortality compared to male patients^3^. Consequently, when doing subgroup analyses to determine whether there are significant differences in in-hospital mortality and other outcome indicators between male and female patients after CABG combined with MVR versus CABG surgery alone, gender should be included as a stratification variable.

Furthermore, in order to gain a more profound understanding of the overall impact of MVR in conjunction with CABG, it is recommended that a comprehensive risk–benefit analysis be incorporated into the research. This analysis should consider all potential advantages of the procedure, such as enhanced cardiac function and symptom relief, as well as every possible disadvantage, such as mortality rates and surgical complications. Besides, the patient’s quality of life, recuperation following surgery, and long-term health results should be taken into account. These are essential elements for developing individualized treatment plans and enhancing clinical judgment. By using this strategy, we can give patients and medical teams more accurate and thorough information to help them make the best treatment decisions. This comprehensive evaluation will facilitate a better knowledge of the trade-offs between the possible advantages and disadvantages of using MVR in conjunction with CABG, ultimately directing patient-centered and more efficient care approaches.

In conclusion, we express our gratitude to the authors for their thorough review of the research on the combination of MVR and CABG in the treatment of moderate IMR, which has provided valuable insights into the field of cardiac surgery. Nonetheless, due to the limited number of studies included and possible bias, there may be certain limitations to the current findings. Future research should encompass a broader patient population and control for potential bias factors to more accurately assess the efficacy and safety of MVR in conjunction with CABG for treating moderate IMR. By doing so, the cardiac surgery community can benefit from a more robust evidence base that informs clinical practice and ultimately contributes to improved patient outcomes.

## Ethical approval

This comment does not involve any original research or patient data. Therefore, ethical approval is not required.

## Consent

This comment does not involve patient or volunteer data, and thus, consent is not applicable.

## Sources of funding

None.

## Conflicts of interest disclosure

The authors declare no conflicts of interest.

## Author contribution

Both authors are responsible for the concept, writing, and review of this comment.

## Research registration unique identifying number (UIN)

This comment does not involve primary research. Therefore, there is no research registration or unique identifying number.

## Guarantor

The authors are the guarantors of this work and assume full responsibility for the conduct of the study and the decision to publish.

## Data availability statement

This comment does not involve the use of research data, and therefore, there is no data availability statement to provide.

## Provenance and peer review

This comment was not commissioned and is the result of the author’s independent analysis and perspective.
